# Modified hollow mesoporous silica nanoparticles as immune adjuvant-nanocarriers for photodynamically enhanced cancer immunotherapy

**DOI:** 10.3389/fbioe.2022.1039154

**Published:** 2022-10-11

**Authors:** Qianru Li, Qianqian Liu, Heli Li, Liyun Dong, Yajie Zhou, Jintao Zhu, Liu Yang, Juan Tao

**Affiliations:** ^1^ Department of Dermatology, Union Hospital, Tongji Medical College, Huazhong University of Science and Technology (HUST), Wuhan, China; ^2^ Key Laboratory of Material Chemistry for Energy Conversion and Storage, Ministry of Education, School of Chemistry and Chemical Engineering, HUST, Wuhan, China; ^3^ Hubei Key Laboratory of Plasma Chemistry and Advanced Materials, School of Material Science and Engineering, Wuhan Institute of Technology, Wuhan, China; ^4^ Division of Child Healthcare, Department of Pediatrics, Tongji Hospital, Tongji Medical College, HUST, Wuhan, China

**Keywords:** photodynamic therapy, hollow mesoporous silica nanoparticles, polyethyleneimine, adjuvant, immunotherapy

## Abstract

Nanomedicine has demonstrated great potential in enhancing cancer immunotherapy. However, nanoparticle (NP)-based immunotherapy still has limitations in inducing effective antitumor responses and inhibiting tumor metastasis. Herein, polyethylenimine (PEI) hybrid thin shell hollow mesoporous silica NPs (THMSNs) were applied as adjuvant-nanocarriers and encapsulated with very small dose of photosensitizer chlorine e6 (Ce6) to realize the synergy of photodynamic therapy (PDT)/immunotherapy. Through PEI etching, the obtained Ce6@THMSNs exhibited enhanced cellular internalization and endosome/lysosome escape, which further improved the PDT efficacy of Ce6@THMSNs in destroying tumor cells. After PDT treatment, the released tumor-associated antigens with the help of THMSNs as adjuvants promoted dendritic cells maturation, which further boosted CD8^+^ cytotoxic T lymphocytes activation and triggered antitumor immune responses. The *in vivo* experiments demonstrated the significant potency of Ce6@THMSNs-based PDT in obliterating primary tumors and inducing persistent tumor-specific immune responses, thus preventing distant metastasis. Therefore, we offer a THMSNs-mediated and PDT-triggered nanotherapeutic system with immunogenic property, which can elicit robust antitumor immunity and is promising for future clinical development of immunotherapy.

## Introduction

The development of cancer immunotherapy is a promising strategy for the next-generation of cancer therapy, as it is based on teaching or exciting the immune system of the body to detect and kill tumor cells (Khalil et al., 2016; Galon and Bruni, 2019). Immuno-therapeutic approaches, including cancer vaccines, (Irvine et al., 2015), cytokine therapy, (Yi et al., 2018), immune-checkpoint blockade therapy (Gubin et al., 2014; Gettinger et al., 2015), and adoptive T cell transfer [e.g., chimeric antigen receptor (CAR)-T cell therapy] (Maude et al., 2014; Golubovskaya, 2017) have achieved remarkable success in the clinic, especially the latter two treatments. However, these methods still have some limitations. While immune-checkpoint blockade therapies have significantly improved the survival rates of patients with many cancer types, the low response rates and severe immune-related adverse events (IrAEs) have restricted their widespread application clinically (Robert et al., 2015; Long et al., 2018; Postow et al., 2018). CAR-T cell therapy has shown notable clinical efficacy against hematological tumors, but has yet to exhibit any considerable impact on solid tumors due to the formation of the immunosuppressive microenvironment, which has inhibited the infiltration and proliferation of CAR-T cells (Lim and June 2017). Therefore, there is an urgent need for a new treatment method that can regulate the immunosuppressive tumor microenvironment, and be applied to a variety of tumors.

**SCHEME 1:**
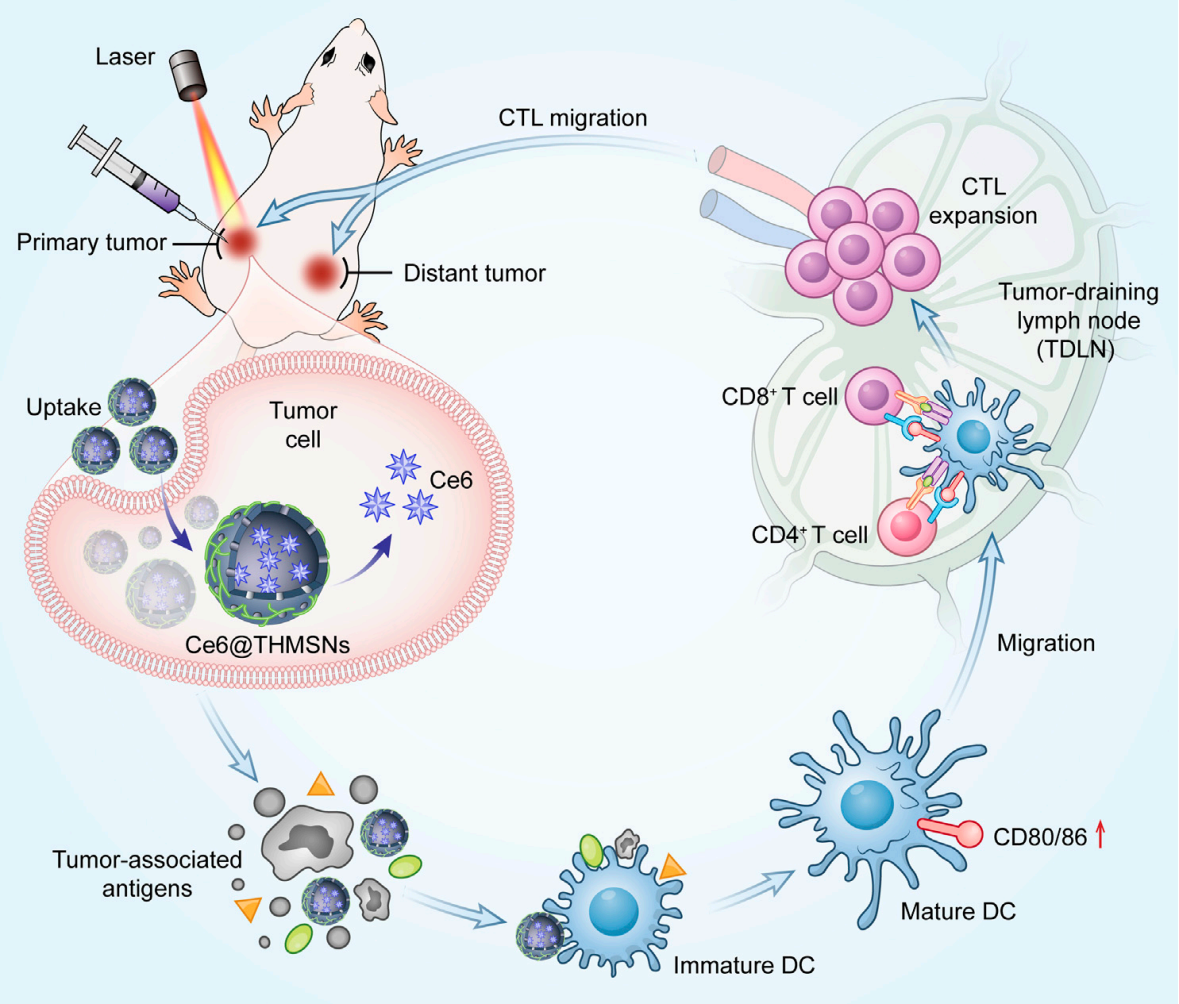
Mechanism of antitumor immune responses induced by Ce6@THMSNs. We introduced PEI hybrid thin shell HMSNs loaded with photosensitizer Ce6 (Ce6@THMSNs) to enhance nanoparticle-based cancer immunotherapy. Ce6@THMSNs-based PDT realized the synergy between Ce6-induced PDT and THMSNs-mediated adjuvant effect, strongly induced antitumor immune responses, enabling the forceful elimination of primary tumors and tumor metastasis. This study may provide a potential agent to improve the efficiency of tumor immunotherapy."

Photodynamic therapy (PDT) has obtained clinical approval and been successfully used against many solid tumors, (Fan et al., 2016, Fan et al., 2017), which can effectively activate antitumor immunity by exciting photosensitizers (PS) with laser irradiation and destroying tumor cells to release tumor-associated antigens (TAA) (He et al., 2016). Especially, PDT has identified to have the significant advantages of less damage to normal tissues and high safety (Huang et al., 2011). However, the limited penetration of laser irradiation makes PDT usually failure in treating distant tumors or tumor metastasis (Zhou et al., 2016). Moreover, PDT-instigated immune responses are typically limited, resulting in its inability to halt the continuous growth of the remaining tumor cells (Castano et al., 2006). Therefore, some attempts have been made to activate stronger immune response through the strategy of combination NPs with adjuvants. Liu et al. constructed poly (lactic-co-glycolic acid) (PLGA) NPs that simultaneously contain a cell-penetrating peptide and two cytokines as adjuvants for effective cancer immunotherapy (Liu et al., 2013). Unfortunately, traditional nanocarriers (e.g., PLGA nanospheres) have low adjuvant loading efficiency and face difficulty exposing adjuvants because of the carrier entrapment (Wang et al., 2018). In summary, a new strategy is urgently needed to directly cause tumor cell death and effectively activate the immune response.

In this study, we fabricated thin-shell polyethyleneimine (PEI)-hybrid hollow mesoporous silica nanoparticles (HMSNs) loaded with photosensitizer chlorine e6 (Ce6) (denoted as Ce6@THMSNs) to realize photodynamic-triggered immunotherapy (Scheme 1). HMSNs with large and uniform pore size, feasibility of surface functionalization and excellent biocompatibility are attractive nanocarriers (Chen et al., 2013; Wang et al., 2022). After PEI etching, we found that the obtained THMSNs could serve as both excellent vehicles and immunoadjuvants, due to the positively charged surface and the thin-shell structures (Liu et al., 2019). Ce6@THMSNs acquired increased cellular uptake and promoted endosome/lysosome escape, thus the PDT efficacy was improved even under a relatively low dose of Ce6. More significantly, Ce6-induced PDT together with THMSNs-mediated adjuvant effect showed vaccine-like functions, efficiently promoting dendritic cells (DCs) maturation and cytotoxic T lymphocytes cells (CTLs) activation. This could boost antitumor immunity, enabling the prevention of tumor progression and distant metastasis. Therefore, we have demonstrated the significant potency of adjuvant THMSNs-based PDT in promoting persistent tumor-specific immune responses and preventing tumor progression, indicating Ce6@THMSNs a potential agent in promoting the efficiency of cancer immunotherapy.

## Experimental section

### Synthesis of thin shell hollow mesoporous silica nanoparticles

THMSNs were synthesized following our previous report. (Liu et al., 2019). Briefly, mesoporous silica coated solid silica nanoparticles (sSiO_2_@mSiO_2_ NPs) with core-shell structure were firstly formed, subsequently, etched for 30 min by sodium carbonate (Na_2_CO_3_, Sinopharm Chemical Reagent Co.) with the concentration of 0.4 M, mesoporous core-shell silica (mCSiO_2_) NPs were obtained after removing CTAB micelles. Finally, the as-prepared mCSiO_2_ NPs were further etched by 2.0 mg/ml of branched PEI (*M*_*w*_ = 25 kDa, Sigma-Aldrich) for 2 h. After removing free PEI, THMSNs were obtained. The morphology, structure, and surface chemistry of THMSNs were investigated using transmission electron microscope (TEM, Tecnai G220, FEI company, Holland), field emission transmission electron microscope (FTEM, Tecnai G2 F30, FEI company, Holland), and dynamic light scattering (DLS, Nano-ZS90, Malvern Instruments Ltd., United Kingdom).

### Preparation of chlorine e6 loaded thin shell hollow mesoporous silica nanoparticles

Ce6@THMSNs were prepared by a solution-solvent evaporation method. 133 μl of Chlorin e6 (Ce6, Frontier Scientific, Inc.) dimethyl sulphoxide (DMSO, Sinopharm Chemical Reagent Co.) solution with the concentration of 1.0 mg/ml was added into 5 mg THMSNs. After mixing by sonication for 10 min, the suspensions in liquid state were evaporated by negative pressure. Ce6@THMSNs were achieved after removing unabsorbed excess Ce6 by washing the deposits. Ce6 loaded HMSNs (Ce6@HMSNs) as control group was synthesis according to the above method. Ce6@THMSNs, Ce6@HMSNs, and Ce6 were evaluated by UV-Vis spectrophotometer (UV-1800, SHIMADZU, Japan). The concentration of Ce6 in the supernatant was analysed by UV-Vis spectrophotometer to measure the drug loading capacity (DL) and encapsulation efficiency (EE) of Ce6. Fluorescence spectrophotometer of the Ce6@THMSNs, Ce6@HMSNs, and Ce6 were analyzed *via* a Fluoromax-4 spectrofluorometer (Horiba Jobin Yvon Inc.) under 400 nm excitation.

### Measurement of singlet oxygen

Singlet oxygen was detected as follows method (Tian et al., 2011). Solutions containing Ce6@THMSNs, Ce6@HMSNs, or Ce6 (0, 1, 2, and 4 μg/ml of Ce6; or 0, 37.5, 75, and 150 μg/ml of HMSNs or THMSNs equivalent) were mixed with N, N-dimethyl-4-nitrosoaniline (RNO, 300 μM, Energy Chemical Inc.), L-Histidine (30 mM, Sigma-Aldrich), and PBS (10 mM, pH = 7.4). Subsequently, the solutions were irradiated for certain time periods under 655 nm laser. The absorption of RNO at 440 nm were bleached by singlet oxygen generated under irradiation, then was detected by the UV-Vis spectrophotometer. The production of singlet oxygen (^1^O_2_) was proved by the diminished optical density at 440 nm.

### Cellular uptake and intracellular distribution

4T1 cells were cultured in a standard environment (37°C and 5% CO_2_). For confocal laser scanning microscopy (CLSM) analyzing, these cells were incubated with Ce6@THMSNs, Ce6@HMSNs, Ce6, or phosphate-buffered saline (PBS) for 6 h using the same 1 µg/ml Ce6 concentration across groups. Nuclei were stained on coverslips using DAPI per the manufacturer’s directions, mounted on microscopic slides, and imaged with a Leica SP5 confocal microscope. To study the intracellular localization of NPs, the cells were incubated with fresh medium containing LysoTracker (100 nM, Invitrogen; 1 h, 37°C). These results were imaged by confocal microscope (Olympus IX73) and the overlap coefficient between the red and green fluorescence signal were calculated by Image-Pro Plus 6.0 software. For flow cytometry evaluations, the cells were incubated with Ce6@THMSNs, Ce6@HMSNs, Ce6, or PBS (the concentration of Ce6 was 1 μg/ml across all groups) at different times and the harvested cells were assessed with flow cytometry (BD LSR-2, United States).

### *In vitro* photodynamic toxicity and dark toxicity

To evaluate photodynamic toxicity, 4T1 cells were stimulated with PBS or Ce6@THMSNs, Ce6@HMSNs, Ce6 with the concentration of Ce6 (1 μg/ml) across all groups at different times or several Ce6 concentrations for 6 h. The cells were subsequently subjected to irradiation from 655 nm laser (MD-655-HS-1.8W, CNIlaser, China) for 1 min (0.3 W/cm^2^). After irradiation, cells were incubated with Cell Counting Kit-8 (CCK-8) solution (Boster, China) for an additional 1 h, and their relative viabilities were determined using a microplate reader (Infinite F50, Tecan Austria, Austria) to read absorbances at 450 nm.

For the dark cytotoxicity study, the cells were incubated with Ce6@THMSNs, Ce6@HMSNs, Ce6, or PBS at various Ce6 concentrations for 6 h and then their relative viabilities were determined using the CCK8 assay, as described above.

### *In vitro* dendritic cell stimulation experiments

Bone-marrow-derived dendritic cells (BMDCs) were obtained from 6-8-week-old BALB/c mice acquired from Beijing Huafukang Bioscience Co., Inc., as detailed in a previous report, (Xie et al., 2017), and co-cultured them with Ce6@THMSNs, Ce6@HMSNs, Ce6, THMSNs, and HMSNs, separately [(THMSNs) = 30 μg/ml, (Ce6) = 1 μg/ml, (HMSNs) = 30 μg/ml] for 24 h. In addition, 10 ng/ml LPS were added into BMDCs to serve as positive control and untreated BMDCs set were used as negative control. At the end of the various treatments, the BMDCs were harvested and incubated with PerCy7-CD11c (Biolegend), PE-CD86 (Biolegend), Brilliant Violet 421™ MHC-II (I-A/I-E) (Biolegend), and FITC-CD80 (Biolegend) antibodies, and assessed with flow cytometry (BD LSR-2, United States). For co-culture system, 4T1 cells were incubated with various nanoparticles for 6 h. After replacement of fresh medium, cells were exposed to 655 nm laser. Then the suspension was collected and added in BMDCs incubating for another 24 h.

### Animal model

The BALB/c mice (6–8 weeks old) were purchased from Beijing Huafukang Bioscience Co., Inc., and mice experiments were performed per Chinese law and approved by the Animal Experimentation Ethics Committee of the Huazhong University of Science and Technology (IACUC Number: 2891). The mice were partitioned randomly into groups. To establish the orthotopic tumor model, 4T1 cells (5 × 10^5^) were suspended in PBS and then subcutaneously injected into the right rear flank of mice. To set up the dual tumor model, 5 × 10^5^ 4T1 cells were subcutaneously injected into the left side of mice for the primary tumor and 5 × 10^5^ 4T1 cells were subcutaneously injected into the right side of the mice 7 days later to create the distant tumor.

### *In vivo* therapeutic efficacy of chlorine e6 loaded thin shell hollow mesoporous silica nanoparticles-based photodynamic therapy

Upon the volumes of tumors reaching about 80 mm^3^, we randomly partitioned the 4T1 tumor bearing mice into 8 groups (*n* = 4) and injected PBS (control), free Ce6, and the various nano-formulations intratumorally at the Ce6 dose of 6 μg, THMSNs dose of 60 μg, HMSNs dose of 60 μg per mouse on day 0, day 3rd, and day 6th. 6 h after the injection, the mice from four selected groups were irradiated by a 655 nm laser for 3 min (0.3 W/cm^2^ power density) and we monitored their tumor sizes [V= (length × width^2^)/2] and body weights every 2 days before sacrificing them at the end of day 12th. We harvested the tumors and major organs, photographed the tumors and weighed and fixed them with 4% PFA for staining with H&E, and Ki-67.

To examine immune cells after the antitumor study, TDLNs and tumors were harvested on day 9th for flow cytometry. The TDLNs were crushed and filtered through a 70 μm strainer and the obtained single cell suspension in each case was washed twice and re-suspended in PBS containing 2% FBS. The single cells in TDLNs were obtained and blocked with anti-CD16/32 (FcBlock) (Biolegend) for 10 min and further incubated with PerCy7-CD11c (Biolegend), PE-CD86 (Biolegend), Brilliant Violet 421™ MHC-II (I-A/I-E) (Biolegend), and FITC-CD80 (Biolegend) for 30 min at 4°C. The harvested tumors were digested with 1500 U/ml collagenase (Sigma) for 30 min and then 70 μm filters were used to get the single-cell suspensions. These cells were stained with PE-CD3 (Biolegend), APC-Cy7-CD8a (Biolegend), PerCP-Cy5.5-CD107a (Biolegend), and FITC-CD4 (Biolegend) antibodies for 30 min and evaluated utilizing flow cytometry. Data were analyzed with FlowJo V10.

### *In vivo* therapeutic efficacy of chlorine e6 loaded thin shell hollow mesoporous silica nanoparticles-based photodynamic therapy against distant tumors

The day after the distant tumors were inoculated, and the mice were partitioned randomly into 6 groups (*n* = 4) and injected with the same parameters of the various NPs, as explained above, into the primary tumors of all the animals intratumorally. 6 h after the injections, three groups were chosen, which were irradiated by a 655 nm laser (0.3 W/cm^2^, 3 min) and the distant tumors were monitored without treatment. At day 14th, the distant tumors and TDLNs were harvested for the assessment of immune cell populations using flow cytometry, as explained above.

### Statistical analysis

GraphPad Prism 6 software (GraphPad Software Inc., United States) were employed for the statistical analyses and all statistical analyses were presented as the mean ± standard deviation (SD) of at least three separate independent tests. One-way ANOVA were performed for quantitative data comparisons between different groups. *p < 0.05, **p < 0.01, and ***p < 0.001 values denoted statistically significant differences.

## Results and discussion

### Generation and characteristics of chlorine e6 loaded thin shell hollow mesoporous silica nanoparticles

**FIGURE 1:**
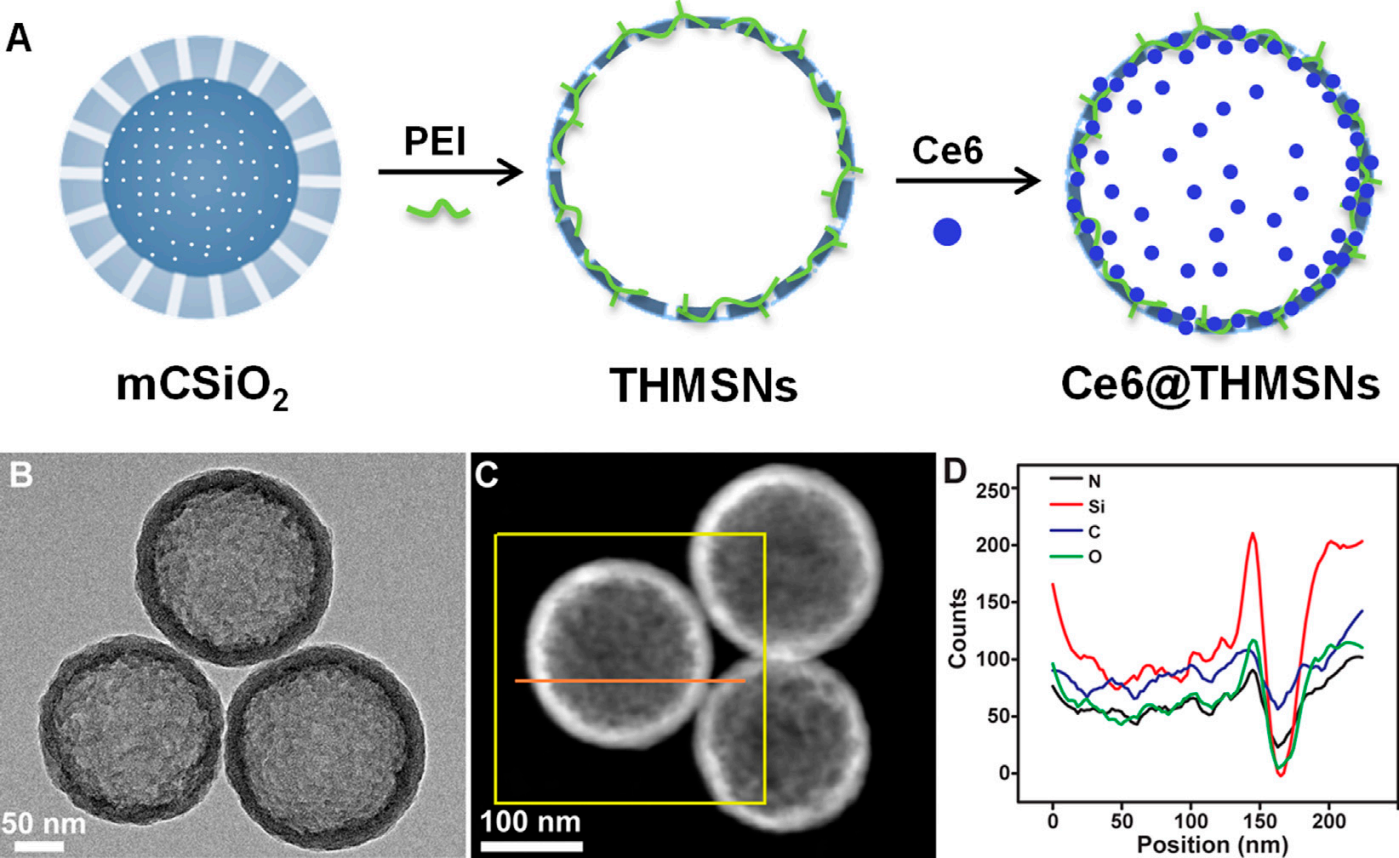
Construction and characterization of Ce6@THMSNs. **(A)** Illustration of the synthesis of Ce6@THMSNs. **(B)** TEM image, **(C)** Dark-field TEM image and **(D)** The EDX of THMSNs.

**FIGURE 2:**
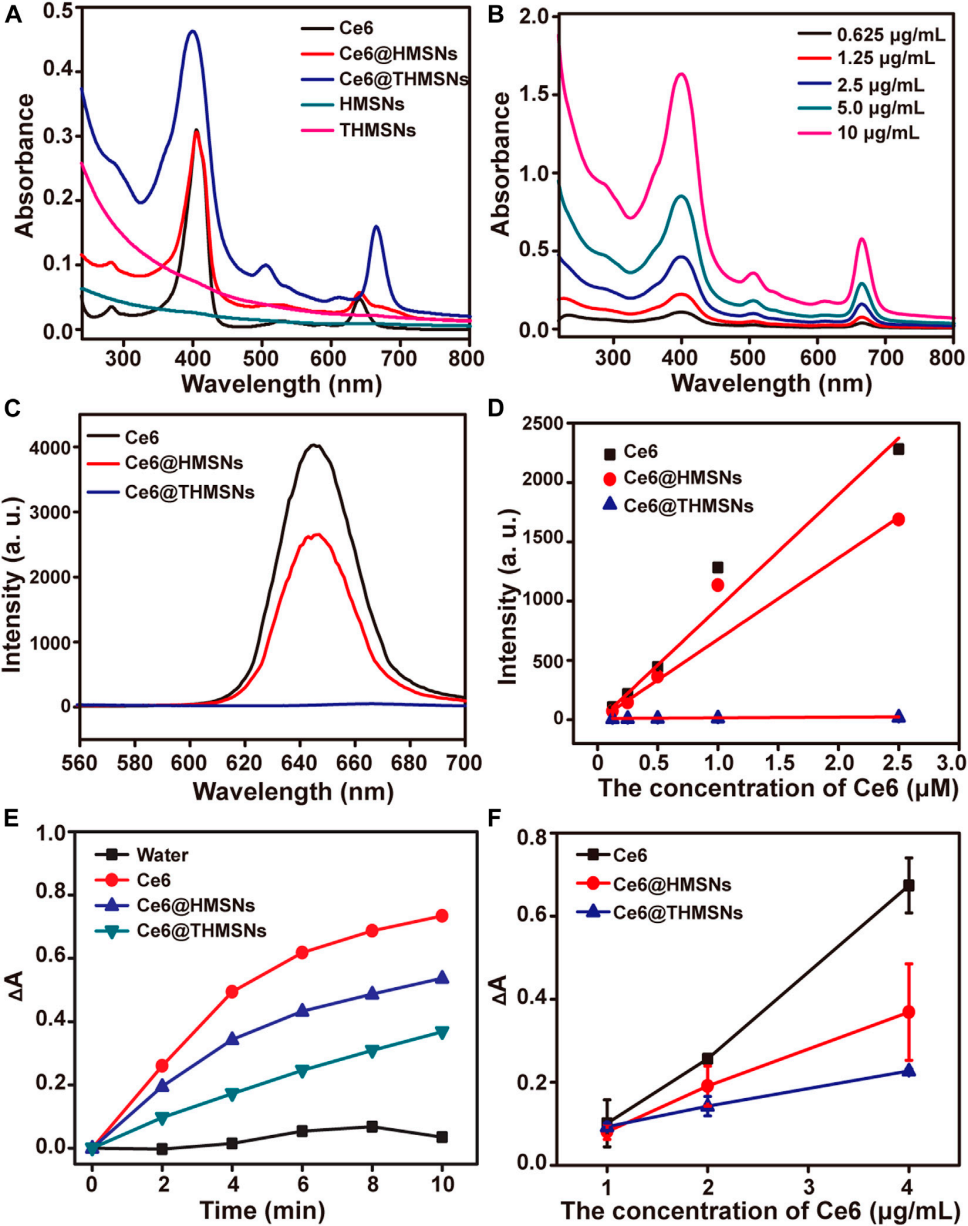
UV-Vis spectra, fluorescence spectra and singlet oxygen generation of nanoformulations and free Ce6. **(A)** UV-vis spectra of Ce6, Ce6@HMSNs, Ce6@THMSNs, HMSNs, and THMSNs. **(B)** UV-vis spectra of Ce6@THMSNs at indicated concentrations of Ce6. **(C)** Fluorescence spectra of Ce6, Ce6@HMSNs, Ce6@THMSNs at 5 μM of Ce6 equivalent under 400 nm excitation. **(D)** Fluorescence spectra of Ce6, Ce6@HMSNs, Ce6@THMSNs at indicated concentration. **(E)** Time-course production of singlet oxygen by Ce6, Ce6@HMSNs, Ce6@THMSNs, HMSNs, and THMSNs at 2.3 μg/ml of Ce6 equivalent under 655 nm laser irradiation. **(F)** Production of singlet oxygen by Ce6, Ce6@HMSNs, and Ce6@THMSNs, at indicated concentrations after 655 nm laser irradiation for 10 min.

THMSNs was synthesized through PEI etching method following our previous report, (Liu et al., 2019), then loaded with Ce6 *via* electrostatic interaction. The illustration of the synthesis of Ce6@THMSNs were shown in Figure 1A. The obtained THMSNs with the size of ∼200 nm had the porous thin shell structure (∼20 nm), as shown in Figures 1B,C. As a control NPs, HMSNs was also prepared, with similar structure (Supplementary Figure S1A) and hydrodynamic size (about 240 nm with PDI of 0.048 and 234 nm with PDI of 0.017 for HMSN and THMSNs, respectively) (Supplementary Figures S1B, S2A). The energy dispersive X-ray spectroscopy (EDX) of THMSNs (Figure 1D) showed that there were consisted of Si, O, N, and C element, which proved that the hybrid PEI and silica in the THMSNs. Ce6 were successfully loaded in the HMSNs and THMSNs, which was confirmed by UV-Vis spectra (Figure 2A). Clearly, there were the characteristic absorption peak of Ce6 at 404 and 641 nm as shown in the UV-Vis spectrum of Ce6@HMSNs. For Ce6@THMSNs, the absorption peak shifted to 400 and 665 nm, respectively, which was ascribed to the interaction of Ce6 and THMSNs. The surface of THMSNs in water was positive and alkaline due to well-known proton-sponge effect of PEI. The absorption was concentration dependent, which increased with the increasing of the concentration of Ce6@THMSNs (Figure 2B). The concentration of Ce6 in HMSNs or THMSNs were also determined by UV-Vis spectrophotometer. The EE and DL of Ce6 for THMSNs were about 97.91% and 16.37%, respectively. Whereas, only reached 76.85% and 8.44% for HMSNs, respectively. Fluorescence spectra of Ce6, Ce6@HMSNs, and Ce6@THMSNs were detected to understand the interaction between silica nanoparticles and Ce6 (Figures 2C,D). It was observed that the about 25% fluorescence of Ce6 loading in HMSNs was quenched, and about 95% for THMSNs, which could be attributed to the tight interaction between Ce6 and THMSNs. The hydrodynamic size of Ce6@THMSNs was about 250 nm with PDI of 0.062 (Supplementary Figure S2B), which demonstrated that the Ce6@THMSNs were well dispersed in water, the slight increase of the hydrodynamic size may due to the drug loaded. All the results above prove the success synthesis of Ce6@THMSNs.

### The generation of singlet oxygen

The production of singlet oxygen (^1^O_2_) was decisive process for PDT. RNO was employed to detect the singlet oxygen, due to its bleaching ability that the absorbance at 440 nm would be diminished triggered by ^1^O_2_ generated by 655 nm laser. As shown in Figures 2E,F, the absorbance of RNO at 440 nm in Ce6, Ce6@HMSNs, and Ce6@THMSNs groups were diminished with different irradiation time and with different concentration of Ce6. Interestingly, the ^1^O_2_ production of Ce6@THMSNs were still substantially increased from 34.8% to 50.1% of ^1^O_2_ generated by free Ce6 with the increase of irradiation time, although the fluorescence of Ce6 loaded in THMSNs were quenched at a certain degree, which contains only about 2% fluorescence of free Ce6. For Ce6@HMSNs, the ^1^O_2_ were substantially generated to 73.2% of that produced by free Ce6 after irradiation 10 min. The highly retained ^1^O_2_ production efficiency of Ce6 loaded on HMSNs or THMSNs and the slow release of ^1^O_2_ possess a promising chance to use Ce6@HMSNs or Ce6@THMSNs for PDT cancer treatment.

### *In vitro* cell internalization and the photodynamic therapy effect of chlorine e6 loaded thin shell hollow mesoporous silica nanoparticles

**FIGURE 3:**
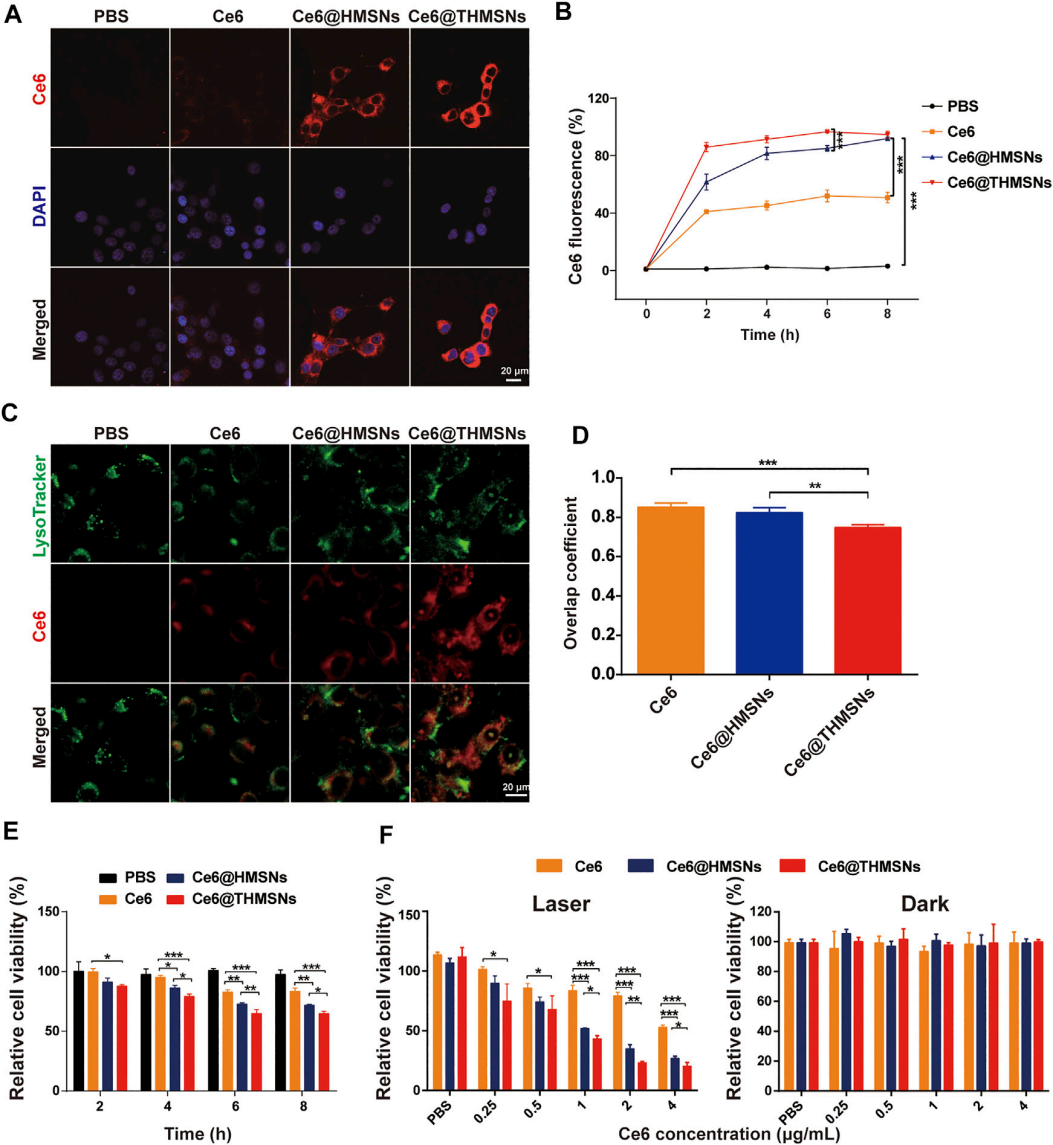
*In vitro* cellular absorption, endolysosomal escape, and the PDT efficiency of Ce6@THMSNs. **(A)** CLSM images of 4T1 cells stimulated by Ce6@THMSNs, Ce6@HMSNs, or Ce6 for 6 h. The nuclei were stained using DAPI (blue). **(B)** Flow cytometry results of the treatment of 4T1 cells with Ce6@THMSNs, Ce6@HMSNs, or Ce6 at different times. **(C)** CLSM images of 4T1 cells stimulated by Ce6@THMSNs, Ce6@HMSNs, or Ce6 for 6 h. Intracellular trafficking was evaluated by staining endosomes/lysosomes using LysoTracker. **(D)** Quantification of the overlap coefficient between lysosome and Ce6, Ce6@HMSNs or Ce6@THMSNs. **(E)** *In vitro* phototoxicities of Ce6@THMSNs, Ce6@HMSNs, and Ce6 in 4T1 cells after co-culture and treatment with irradiation from a 655 nm laser (0.3 W/cm^2^, 1 min) at different times, as determined using the CCK-8 assay. **(F)** 4T1 cell viability after treatment with Ce6@THMSNs, Ce6@HMSNs, and Ce6 at various concentrations with and without irradiation from a 655 nm laser (0.3 W/cm^2^, 1 min), as determined using the CCK-8 assay. Data are presented as the mean ± SD (***p < 0.001, **p < 0.01, or *p < 0.05).

The efficacy of PDT depends on particle internalization. Therefore, we studied the cellular uptake profile of Ce6@THMSNs carefully. We firstly used CLSM to observe an obvious intra-cytoplasm fluorescence pattern of Ce6 in 4T1 cells after stimulated by Ce6@THMSNs and Ce6@HMSNs. In contrast, only weak fluorescence signals of Ce6 were noted in cells incubated with free Ce6 (Figure 3A). Next, using flow cytometry, we confirmed a time-dependent absorption of Ce6@THMSNs by cells. Compared with Ce6 treatment groups, almost all 4T1 cells exhibited remarkable intracellular Ce6 fluorescence after incubation with Ce6@THMSNs and Ce6@HMSNs from 0 to 8 h, indicating that more Ce6@THMSNs and Ce6@HMSNs were taken up intracellularly than free Ce6 (Figure 3B). HMSNs are considered as the representative inorganic NPs for PS delivery in applications of PDT, because of their efficient storage and enhanced intracellular uptake effect (Fan et al., 2017; Vankayala and Hwang, 2018). Our results confirm that Ce6@THMSNs (***p < 0.001), even better than Ce6@HMSNs, could be efficiently internalized by cells, which was an effective nanoplatform for killing tumor cells with PDT.

To determine the manner of intracellular trafficking, we used LysoTracker to identify luminescent colocations in 4T1 cells, which was observed with CLSM. We found that THMSNs encapsulation not only increased the cellular internalization of Ce6 but also transformed the subcellular co-placement of the nanoparticles with lysosomes. As shown in Figures 3C,D, strong Ce6 fluorescence was observed in Ce6@THMSNs group, and the overlap coefficient for Ce6 (green) and lysosomes (red) was 0.74 ± 0.01 (*n* = 3), which was lower than that in the cells incubated with Ce6@HMSNs (0.82 ± 0.02) (*n* = 3). In contrast, fluorescence staining with LysoTracker proved weak Ce6 fluorescence colocalization in free Ce6 group. These results indicate that Ce6@THMSNs promoted endosome/lysosome escape, possibly through incorporating of PEI-mediated “proton sponge effect” occurring in acidic lysosomes (Merdan et al., 2002; Akinc et al., 2005; Shen et al., 2017). This would result in the bursting of the lysosomal membrane and the release of endocytosed Ce6 into the cytosol, improving the efficacy of PDT.

We investigated the PDT efficacy of Ce6@THMSNs next using cell counting kit-8 (CCK-8) cytotoxicity assay. As shown in Figure 3E, Compared to Ce6@HMSNs and free Ce6, Ce6@THMSNs significantly decreased the cell viability after incubation with 4T1 cells for 4 h and then exposure to 655 nm laser (0.3 W/cm^2^, 1 min). When the incubation time was up to 6 h, the PDT efficacy of Ce6@HMSNs increased to higher level, but was still lower than Ce6@THMSNs (**p < 0.01). Based on these observations, our pretreatment of 4T1 cells with Ce6@THMSNs for 6 h showed excellent phototoxicities, which were much higher than that in Ce6@HMSNs (*p < 0.05) and free Ce6 group (***p < 0.001) under the same Ce6 concentration (1 μg/ml) after irradiation with 655 nm laser (0.3 W/cm^2^, 1 min) (Figure 3F). All assessed NP types, including free Ce6, Ce6@HMSNs, and Ce6@THMSNs, exacted no notable toxic effects in cells in the absence of laser irradiation, even under high concentrations (Figure 3F). This demonstrated that Ce6@THMSNs showed high biocompatibility *in vitro*. The superior PDT performance of Ce6@THMSNs over Ce6@HMSNs and free Ce6 probably stemmed from its more efficient intracellular uptake and the “proton sponge effect” enabling lysosome escape. Therefore, Ce6@THMSNs have great potential as a biocompatible nano-platform for effective PDT.

### Chlorine e6 loaded thin shell hollow mesoporous silica nanoparticles-induced *in vitro* dendritic cell maturation

**FIGURE 4:**
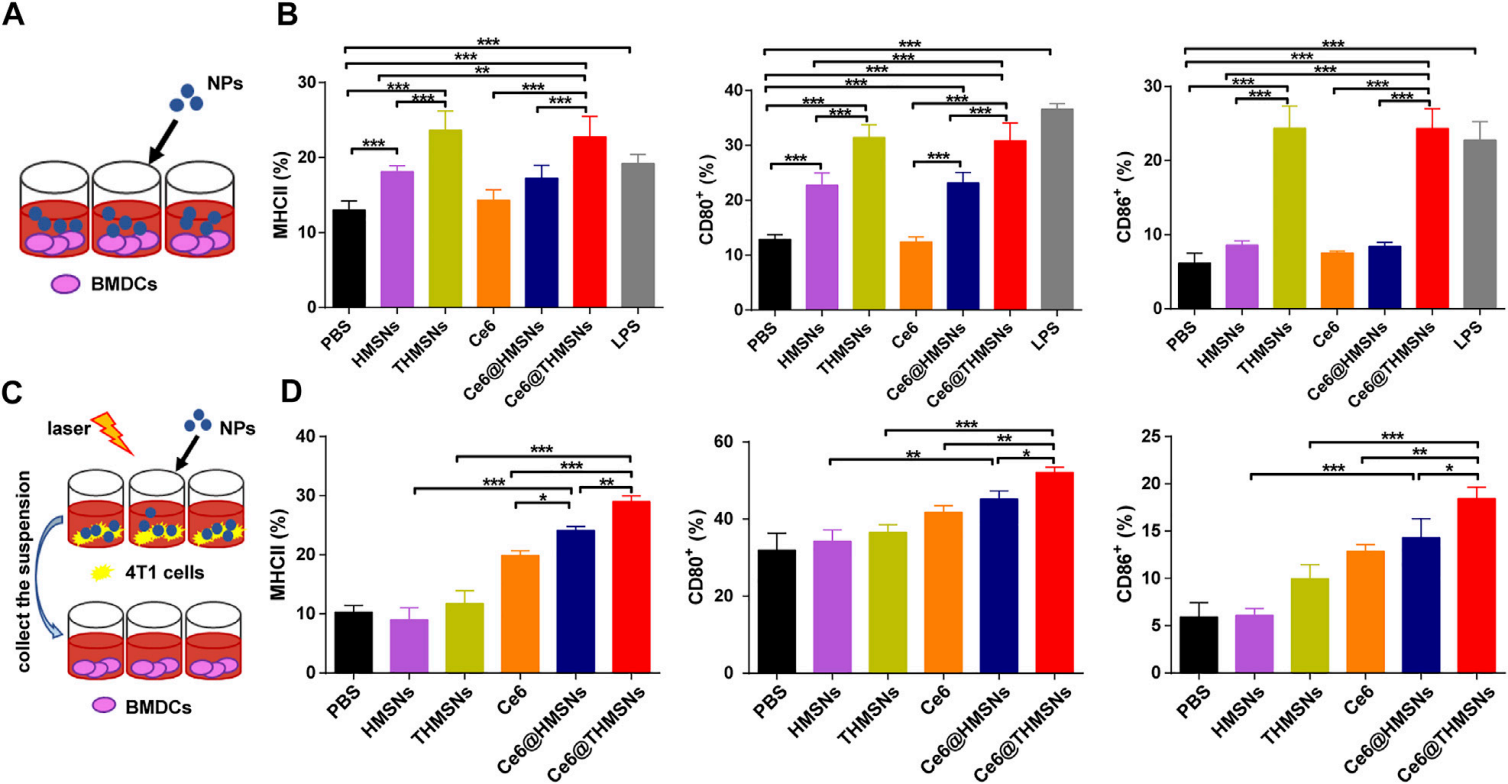
The *in vitro* immune stimulation impact of Ce6@THMSNs. **(A)** Scheme of our experiment for incubating DCs with various NPs. **(B)** Flow cytometry analysis of MHCⅡ, CD80, and CD86 expressions in CD11c^+^ cells, which are indicators of DC maturation, after 24 h of incubation with Ce6@THMSNs, Ce6@HMSNs, Ce6, THMSNs, and HMSNs, respectively. **(C)** Scheme of our experiment measurement of DC maturation after PDT treatment. Collection of the suspension of various NPs incubated 4T1 cells after PDT treatment, which were then added in BMDCs. **(D)** Quantification of MHCⅡ, CD80, and CD86 expressions in CD11c^+^ cells by flow cytometry. Data are presented as the mean ± SD (***p < 0.001, **p < 0.01, or *p < 0.05).

DCs, as the most effective antigen-presenting cells (APCs), can present antigens for the activation of naïve T cells (Banchereau et al., 2000). DC maturation is essential to eliciting an effective immune response. Mature DC phenotypes are marked by the upregulation of major histocompatibility complex classes I/II (MHC-I/MHC-II) and co-stimulatory molecules (CD80 and CD86) (Gardner and Ruffell, 2016). As indicated in Figure 4A, we assessed Ce6@THMSNs’ ability to induce the maturation of immature BMDCs from BALB/c mice. As expected, THMSNs (23.63% ± 2.59%, 31.42% ± 2.34%, 24.35% ± 3.01%) induced notably higher MHCⅡ^+^/CD80^+^/CD86^+^ expression of CD11c^+^ DCs than HMSNs (18.12% ± 0.80%, 22.70% ± 2.29%, 8.58% ± 0.62%) (Figure 4B), which was consistent with findings from our previous research, suggesting the stronger immune stimulation effect of THMSNs than HMSNs (Liu et al., 2019). Similarly, the percentages of the MHCⅡ^+^/CD80^+^/CD86^+^ cells of CD11c^+^ DCs were significantly increased after treatment with Ce6@THMSNs (22.76% ± 2.72%, 30.82% ± 3.22%, 24.34% ± 2.66%) than Ce6@HMSNs (17.24% ± 1.73%, 23.12% ± 1.93%, 8.42% ± 0.55%), with free Ce6 (14.32% ± 1.39%, 12.38% ± 0.94%, 7.52% ± 0.26%) having no impact on DC maturation (Figure 4B). And no significant difference of immuno-stimulating activity of BMDCs was observed in Ce6@THMSNs or THMSNs group. Taken together, the promotion of DC maturation could be attributed to the adjuvant efficacy of THMSNs, pointing to Ce6@THMSNs as powerful immunologic adjuvants.

TAA released after PDT treatment may augment immune responses by taking advantage of immunologic adjuvants (Firczuk et al., 2011). We conducted a co-culture system to analyze such effect *in vitro.* The suspension was collected after various NPs incubated 4T1 cells followed by 655 nm laser irradiation (0.3 W/cm^2^, 1 min), which were used to stimulate BMDCs (Figure 4C). After 24 h incubation, DC maturation was assessed by flow cytometry. Compared to the direct immunostimulatory effect of THMSNs and HMSNs, THMSNs and HMSNs in Figure 4D and Supplementary Figure S3 poorly triggered DC maturation, which could be caused by little released TAA in the absence of destroyed tumor cells. On the other hand, all the groups with good PDT performance (Ce6, Ce6@THMSNs and Ce6@THMSNs) showed significant promotion of DC maturation. Particularly, Ce6@THMSNs (28.97% ± 1.01%, 52.13% ± 1.36%, 18.43% ± 1.22%) showed significant promotion of MHCⅡ^+^/CD80^+^/CD86^+^ expression of CD11c^+^ DCs than Ce6@HMSNs (24.10% ± 0.70%, 45.20% ± 2.12%, 14.30% ± 2.01%) and free Ce6 (19.87% ± 0.80%, 41.80% ± 1.71%, 12.87% ± 0.70%). Taken together, Ce6@THMSNs-based PDT promoted emission of TAA from tumor debris after irradiation, and with the help of THMSNs’ immune adjuvant effect, could boost maturity of DC.

### Chlorine e6 loaded thin shell hollow mesoporous silica nanoparticles-based photodynamic therapy *in vivo* triggered immune responses for antitumor therapy

**FIGURE 5:**
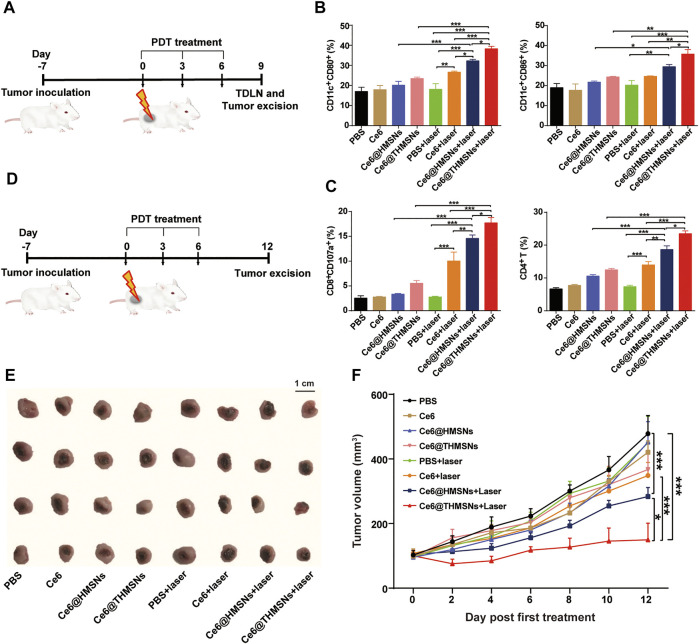
Antitumor immune responses and therapeutic efficacy of Ce6@THMSNs-based PDT *in vivo*. **(A)** The graphic of our experimental procedure to assess antitumor immunity induced by Ce6@THMSNs with PDT treatment. **(B)** Flow cytometry examination of CD11c^+^CD80^+^ and CD11c^+^CD86^+^ cells in TDLNs after three rounds of treatments on day 9th. **(C)** CD8^+^CD107a^+^ T cell and CD4^+^ T cell percentages in tumors after three rounds of treatments on day 9th. **(D)** The graphic of our experimental procedure to assess the therapeutic efficacy of Ce6@THMSNs with PDT treatment. **(E)** Photograph of excised tumors on day 12th. **(F)** The representative curve of tumor volume in different groups after three treatment rounds. Data are presented as the mean ± SD (*n* = 4) (***p < 0.001, **p < 0.01, or *p < 0.05).

Encouraged by the PDT performance and promoted BMDC activation *in vitro*, we further evaluated *in vivo* immune responses and the therapeutic effect of Ce6@THMSNs on BALB/c mice bearing 4T1 tumors. Upon the growth of the 4T1 tumors reaching 80 mm^3^, we separated mice arbitrarily into 8 groups (*n* = 4): 1) PBS, 2) Ce6, 3) Ce6@HMSNs, 4) Ce6@THMSNs, 5) PBS + laser, 6) Ce6 + laser, 7) Ce6@HMSNs + laser, and 8) Ce6@THMSNs + laser. Tumor regions in groups 5, 6, 7, and 8 were irradiated by 655 nm laser (0.3 W/cm^2^, 3 min) at 6 h post-intratumoral administration. The dose for the intratumoral injection of Ce6 16 µg (0.8 mg/ml) in all Ce6 formulations was much lower than that used in other studies on photodynamic immunotherapy (Xu et al., 2017; Yang et al., 2017; Wang et al., 2020). After three rounds of treatments (day 9th), tumors and tumor-draining lymph nodes (TDLNs) were excised for the estimation of the activation of immune responses *in vivo* (Figure 5A). In TDLNs, mice that received PDT with Ce6@THMSNs (38.30% ± 2.76%, 35.80% ± 4.45%) generated much higher rates of DC maturation (CD11c^+^ CD80^+^/CD11c^+^ CD86^+^) than those that received PDT with Ce6@HMSNs (32.26% ± 1.74%, 29.46% ± 2.53%), free Ce6 (26.66% ± 1.18%, 24.70% ± 0.36%), or NP injections without laser irradiation (Figure 5B; Supplementary Figure S4). Antigen-bearing DCs in TDLNs play essential roles in the activation of CD8^+^ cytotoxic T lymphocytes (CTLs) (e.g., CD8^+^CD107a^+^ T cells) and CD4^+^ T helper lymphocytes to produce antitumor immune responses. (Sabado and Bhardwaj, 2015; Giovanelli et al., 2019). Here, Ce6@THMSNs-based PDT therapy elicited considerably higher levels of CD8^+^CD107a^+^ T lymphocytes (17.73% ± 2.16%) and CD4^+^ T lymphocytes (23.48% ± 1.94%) in tumors compared to the other treatment approaches (Figure 5C; Supplementary Figure S5), suggesting that Ce6@THMSNs-based PDT effectively enhanced the proliferation of antigen-specific T cells. Overall, after PDT treatment, tumor-derived antigens from tumor cell debris were released and processed by DCs to initiate antitumor immune responses, during which Ce6@THMSNs acted as immunoadjuvants to amplify immunoreactivity.

Having demonstrated obvious antitumor T cell immunological responses mediated by Ce6@THMSNs + PDT treatment, we examined their effects in inhibiting tumor growth (Figure 5D). Tumor volumes were recorded every other day after the first PDT treatment. On day 12th, we collected tumors and photographed them (Figure 5E). As the tumor growth curves showed (Figure 5F), rapid tumor growth was observed in the PBS and PBS + laser groups. In parallel, mice treated with Ce6, Ce6@HMSNs, or Ce6@THMSNs but without exposure to irradiation displayed no efficient suppression of tumor growth. Nevertheless, single PDT treatment had no significant suppressive impact on tumors, as shown in the free Ce6 + laser group. Compared to the PBS group, Ce6@HMSNs (***p < 0.001) and Ce6@THMSNs (***p < 0.001) with laser irradiation showed better inhibition properties due to their superior PDT effect and PDT-induced TAA production, alongside proper adjuvant, resulting in enhanced therapeutic efficacy. Specially, Ce6@THMSNs + laser treatment drastically suppressed tumor development than Ce6@HMSNs + laser group (*p < 0.05), demonstrating the exceptional adjuvant efficacy of THMSNs. Additionally, tumor photography captured the smallest tumor volumes in the Ce6@THMSNs + laser group on day 12th, further establishing the superior antitumor property of Ce6@THMSNs (Figure 5E).

### Histological analysis of antitumor efficacy and the biocompatibility of chlorine e6 loaded thin shell hollow mesoporous silica nanoparticles

**FIGURE 6:**
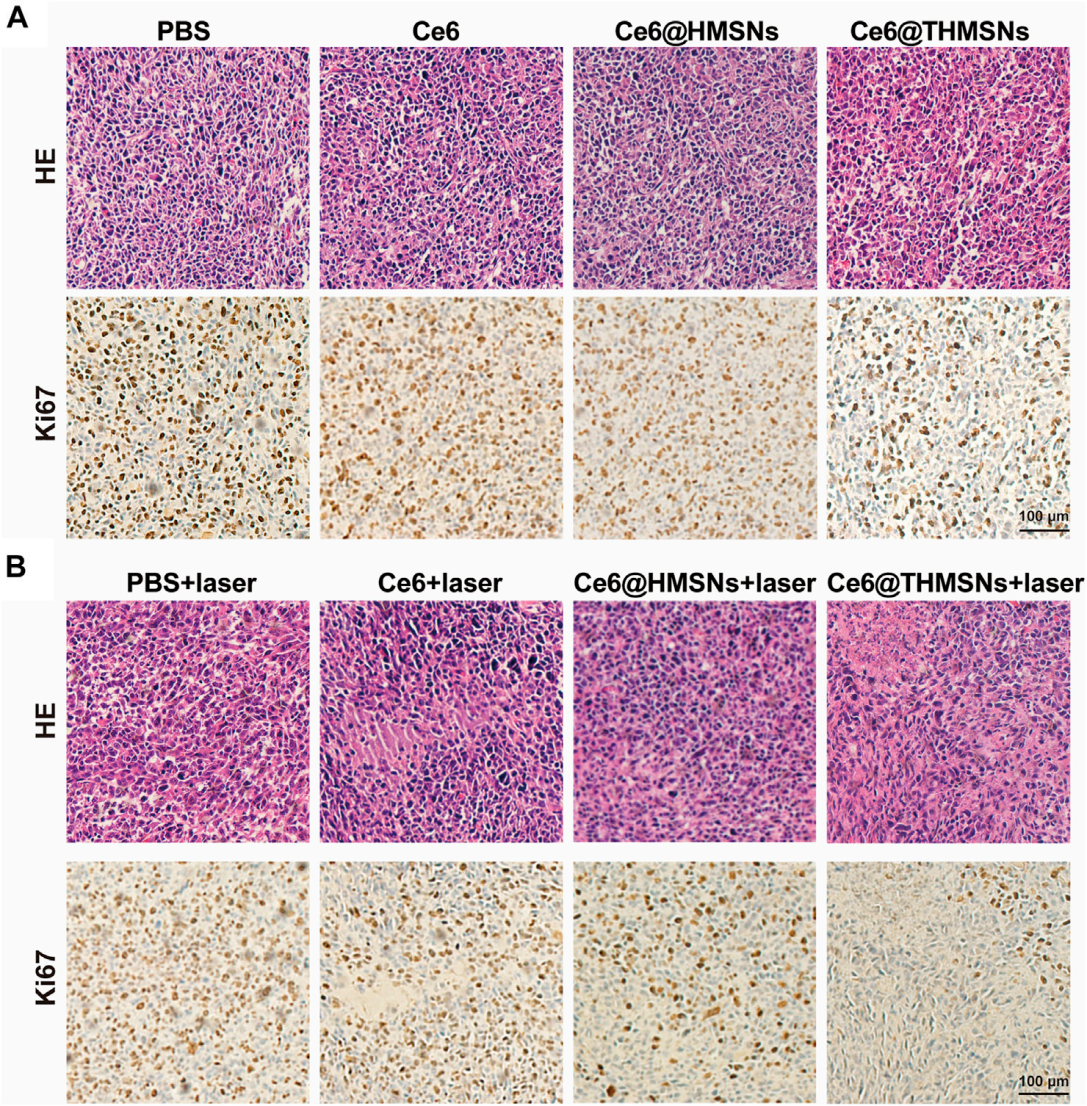
The histological analysis of the antitumor effect and *in vivo* biocompatibility of Ce6@THMSNs. **(A,B)** Representative images of staining tumor sections collected on day 12th with H&E and Ki67. Scale bars: 100 μm.

At the end of the above experiments (day 12th), hematoxylin and eosin (H&E) and Ki67 were employed to evaluate the antitumor efficacy of the various treatment options. As shown in Figures 6A,B, all groups with laser irradiation, except PBS + laser group, displayed cellular damage. Enhanced tumor tissue necrosis was also noted in the Ce6@THMSNs + laser group. Concurrently, Ki67 immunohistochemistry (IHC) staining results registered the lowest level of tumor cell proliferation in the Ce6@THMSNs + laser group, pointing to Ce6@THMSNs’ enhancement of therapeutic properties. Interestingly, in the absence of laser irradiation, Ce6@THMSNs exhibited a decrease in cancer cell proliferation than other groups under laser off conditions, further confirmed the enhanced adjuvant effect of THMSNs (Supplementary Figure S6). Furthermore, we probed the toxicity and systematic side effect of Ce6@THMSNs *in vivo*. No notable tissue damage or inflammatory cell infiltration was observed in major organs (heart, liver, spleen, lung, and kidney) from all groups after treatment (Supplementary Figure S7). Ce6@THMSNs is, therefore, a promising nanoplatform with no evident systemic toxicity *in vivo*.

### Chlorine e6 loaded thin shell hollow mesoporous silica nanoparticles-based photodynamic therapy *in vivo* for inhibiting distant tumors

**FIGURE 7:**
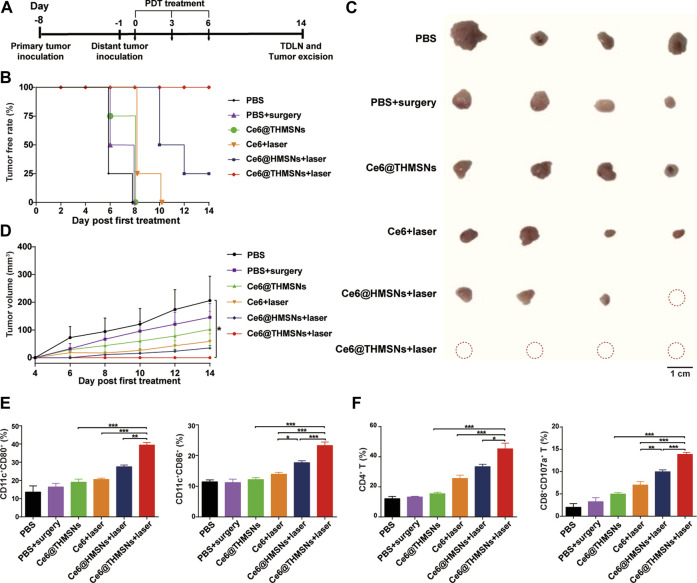
Antitumor efficacy against distant tumors and the underlying mechanism triggered by Ce6@THMSNs-based PDT. **(A)** The graphic of our bilateral tumor model. **(B)** The tumor free rate of distant tumors without treatment. **(C)** Image of excised distant tumors on day 14th. **(D)** Tumor volume curve of distant tumors. **(E)** Flow cytometry examination of CD11c^+^CD80^+^ and CD11c^+^CD86^+^ cells in the TDLNs of distant tumors on day 14th. **(F)** CD4^+^ T cells and CD8^+^CD107a^+^ T cells percentages in the TDLNs of distant tumors on day 14th. Data are presented as the mean ± SD (*n* = 4) (***p < 0.001, **p < 0.01, or *p < 0.05).

Next, we explored the ability of Ce6@THMSNs + PDT-triggered antitumor immune response to inhibit distant tumors untreated with PDT. The design of our dual tumor model was shown in Figure 7A. 7 days after the inoculation of 5 × 10^5^ 4T1 cells in the left flank (the primary tumor) of BALB/c mice, we injected the same amount of 4T1 cells into the right flank (the distant tumor) of the mice and partitioned them randomly into 6 groups (*n* = 4): 1) PBS, 2) PBS + surgery, 3) Ce6@THMSNs, 4) Ce6 + laser, 5) Ce6@HMSNs + laser, and 6) Ce6@THMSNs + laser. In the following day, the primary tumors of all the mice were subjected to the same parameters of the various NPs mentioned above, and 6 h later, the primary tumors from mice in groups 4, 5, and 6 were exposed to 655 nm laser (0.3 W/cm^2^, 3 min), whereas, the distant tumors were monitored without treatment. All the mice received three rounds of treatment. On day 6th, the primary tumors in group 2 were removed through surgery after intratumoral injections of PBS (20 μl) for three rounds. Mice treated with Ce6 + laser or Ce6@HMSNs + laser later harbored untreated, distant tumors on day 8th and 10th, while distant tumors in groups without PDT treatment appeared as early as day 6 (Figure 7B). These results demonstrated how essential PDT inhibited tumor growth through boosting antitumor immunity. Remarkably, Ce6@THMSNs + laser completely suppressed distant tumor growth compared to other groups (Figures 7B–D). In contrast, The Ce6 + laser group or Ce6@HMSNs + laser group did not effectively hinder the occurrence and growth of distant tumors, highlighting the indispensable role of THMSNs in our system as adjuvant to amplify immune response.

To appreciate the mechanism of antitumor efficacy induced by Ce6@THMSNs-based PDT, we examined immune cells from the TDLNs of distant tumors on day 14th (8 days after the final PDT irradiation). Ce6@THMSNs-based PDT treatment prompted significant upregulation of CD80 (39.48% ± 2.36%) and CD86 (23.29% ± 2.25%) among DCs in the TDLNs of distant tumors compared to the other treatment groups (Figure 7E). Meanwhile, we scrutinized subtypes of T lymphocytes for Ce6@THMSN-based PDT’s ability to trigger effective cellular immune response. For mice whose primary tumors were treated with Ce6@THMSNs + laser, the rates of CD8^+^ CTLs and CD4^+^ T cells (13.88% ± 0.85%, 45.34% ± 7.49%) in the TDLNs of distant tumors were more significant than Ce6@THMSNs group (4.97% ± 0.76%, 15.52% ± 1.47%) (Figure 7F), indicating that Ce6@THMSNs-based PDT activated cellular immunity and enhanced CTL recruitment and infiltration into tumor (Mitchell et al., 2015; Seyfizadeh et al., 2016). Collectively, these results suggested that Ce6@THMSNs-based PDT induced TAA releases, DC maturation, and subsequently T cells activation, which can attack tumor instantly.

## Conclusion

In this study, we demonstrated an immunogenic Ce6@THMSNs nanotherapeutic system with efficient PDT performances for cancer immunotherapy. The Ce6@THMSNs act as both an effective vehicle for Ce6 and promising immunoadjuvant itself. Moreover, incorporating of PEI can not only enhance cellular internalization, promote endosome/lysosome escape to produce potent PDT efficacy, but also upregulate the maturity of DC to trigger antitumor immunity. Thus, Ce6@THMSNs-based PDT realized the synergy between Ce6-induced PDT and THMSNs-mediated adjuvant effect, strongly induced antitumor immune responses, thus enabling the forceful elimination of primary tumors and tumor metastasis with very small dose of Ce6. Therefore, we provided a powerful synergistic strategy of Ce6@THMSNs-based PDT for cancer immunotherapy. Their application provided a promising theoretical basis for future clinical development of immunotherapy strategies.

## Data Availability

The original contributions presented in the study are included in the article/Supplementary Material, further inquiries can be directed to the corresponding authors.

## Abbreviations

APCs, antigen-presenting cells; BMDCs, Bone-marrow-derived dendritic cells; CAR, chimeric antigen receptor; Ce6, chlorine e6; CTLs, cytotoxic T lymphocytes cells; CLSM, confocal laser scanning microscopy; DCs, dendritic cells; DL, drug loading capacity; DLS, dynamic light scattering; DMSO, dimethyl sulphoxide; EDX, energy dispersive X-ray spectroscopy; EE, encapsulation efficiency; FTEM, field emission transmission electron microscope; H&E, hematoxylin and eosin; HMSNs, hollow mesoporous silica nanoparticles; IHC, immunohistochemistry; IrAEs, immune-related adverse events; PBS, phosphate-buffered saline; PDT, photodynamic therapy; PEI, polyethylenimine; PLGA, poly (lactic-co-glycolic acid); PS, photosensitizers; mCSiO2, mesoporous core-shell silica; NP, nanoparticle; TAA, tumor-associated antigens; TEM, transmission electron microscope; THMSNs, thin shell hollow mesoporous silica nanoparticles.
