# Supplementary material for: Modified hollow mesoporous silica nanoparticles as immune adjuvant-nanocarriers for photodynamically enhanced cancer immunotherapy
Source: Front Bioeng Biotechnol. 2022 Oct 11;10:1039154. doi: 10.3389/fbioe.2022.1039154 (PMC9592702; doi:10.3389/fbioe.2022.1039154)
Supplement: Supplementary file 1 [file DataSheet1.docx]

Supplementary Material

# Supplementary Figures


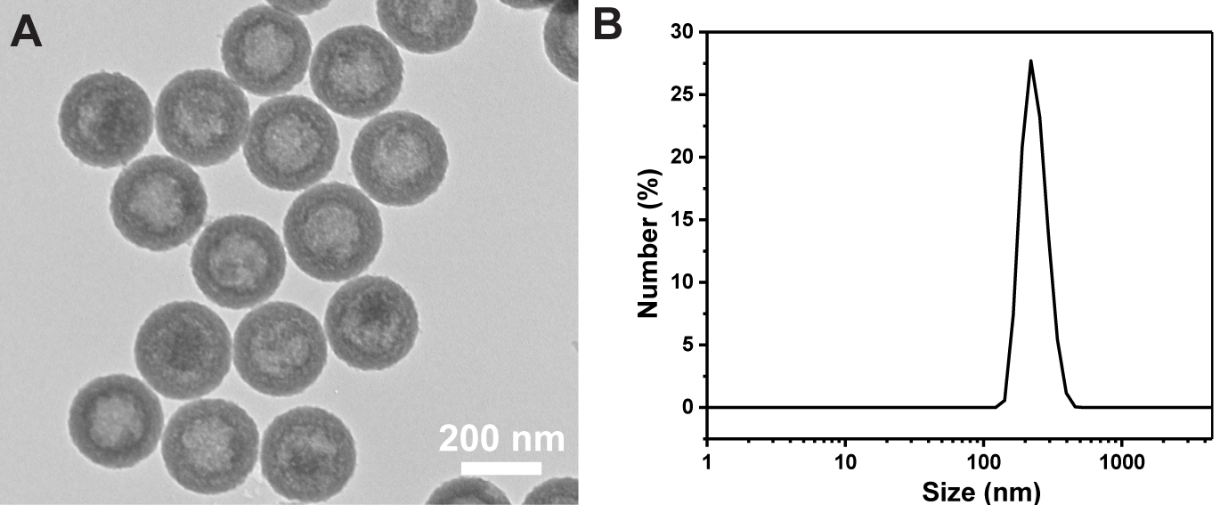


**Supplementary Figure 1.** The (A) TEM image and (B) the hydrodynamic size of HMSNs.


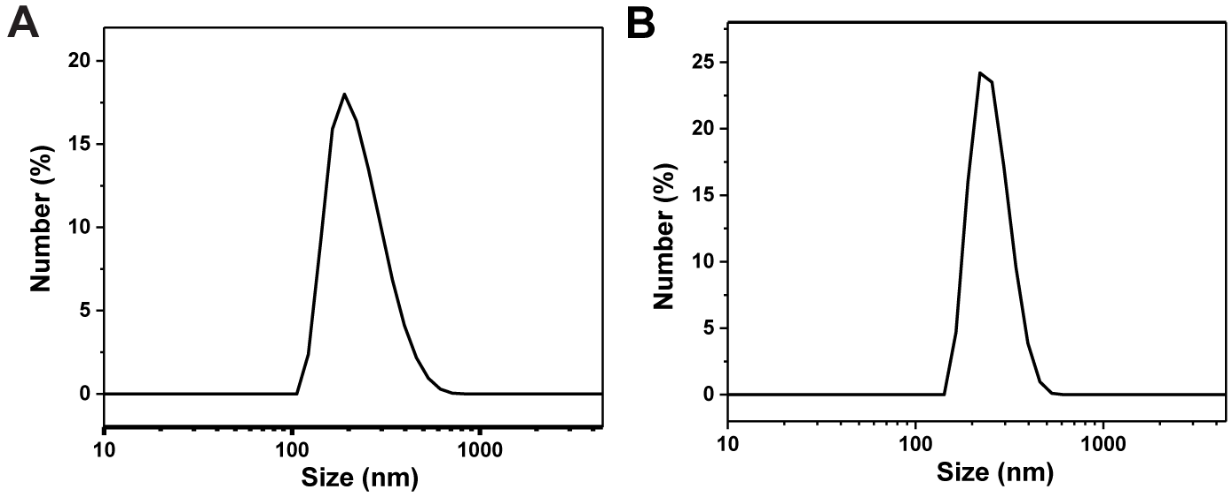


**Supplementary Figure 2.** The hydrodynamic size of THMSNs and Ce6@THMSNs.


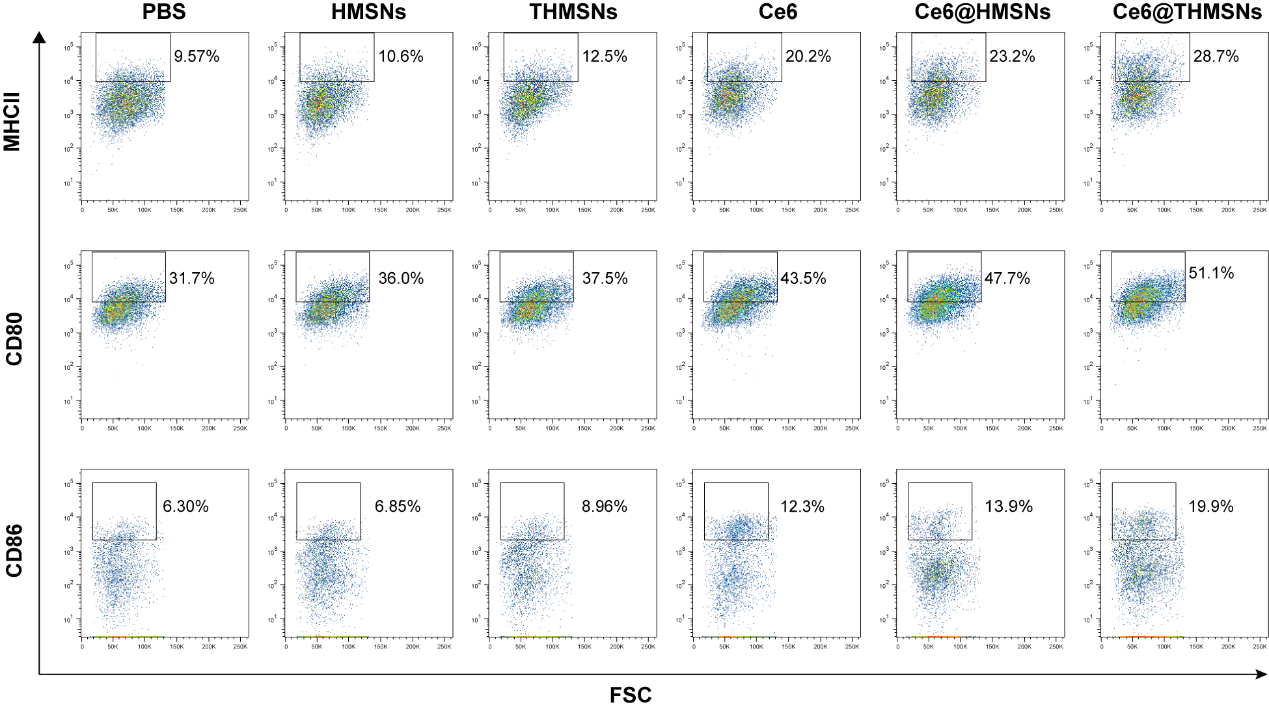


**Supplementary Figure 3.** Quantification of MHCⅡ, CD80, and CD86 expressions in CD11c^+^ cells by flow cytometry after PDT treatment.


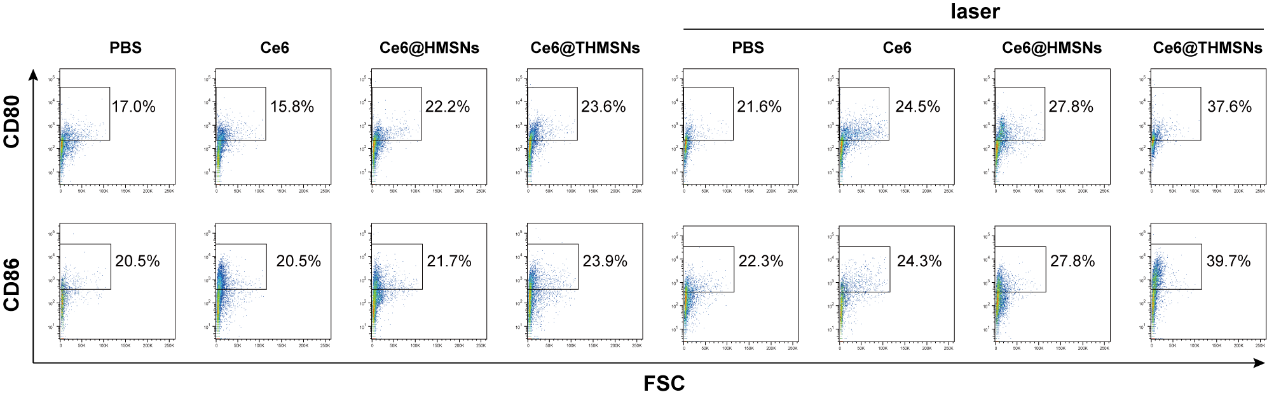


**Supplementary Figure 4.** CD11c^+^CD80^+^ and CD11c^+^CD86^+^ cells in TDLNs after three rounds of treatments by flow cytometry.


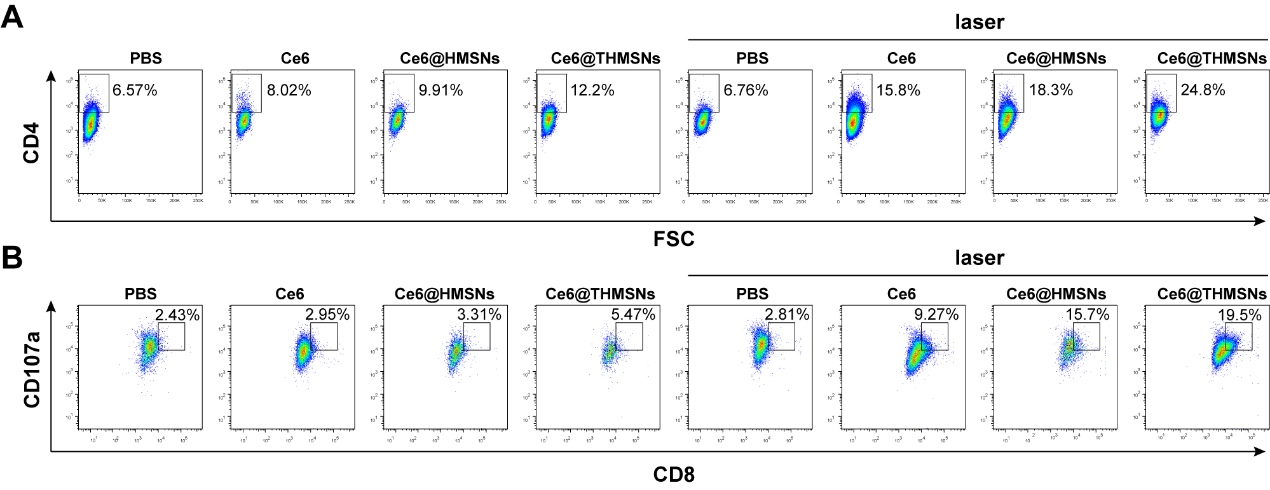


**Supplementary Figure 5.** (A) CD4^+^ T cell and (B) CD8^+^CD107a^+^ T cell percentages in tumors after three rounds of treatments by flow cytometry.


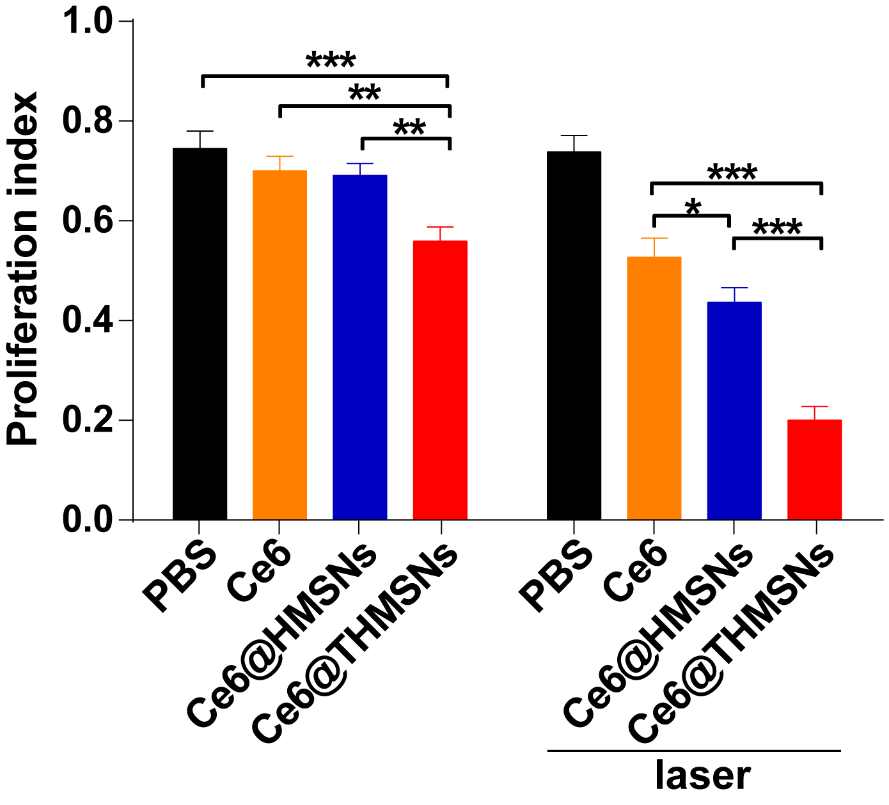


**Supplementary Figure 6.** Proliferative cells (Ki67) quantification in tumor sections after treatment.


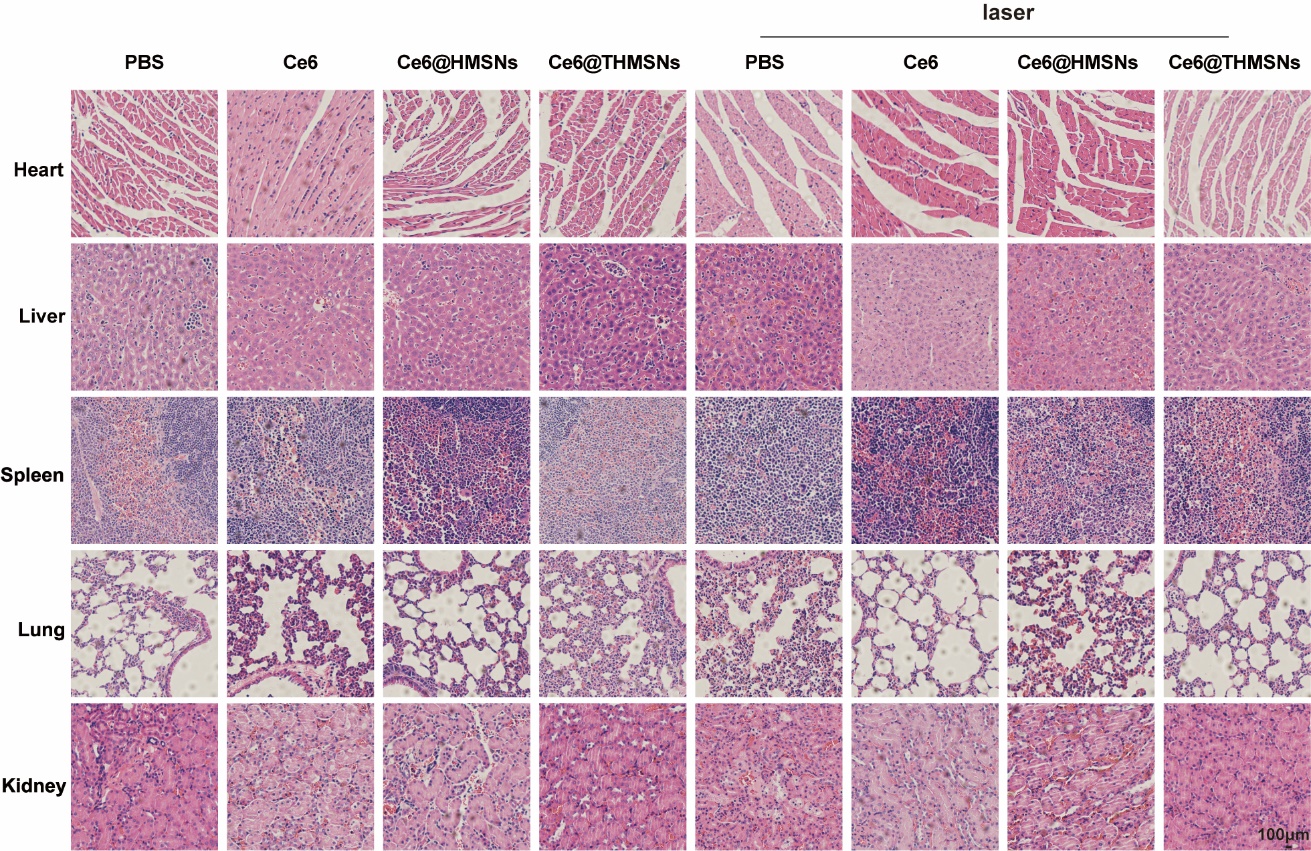


**Supplementary Figure 7.** Results of staining the heart, liver, spleen, lung, and kidney collected on day 12^th^ with H&E.
